# A critical race analysis of structural and institutional racism: Rethinking overseas registered nurses' recruitment to and working conditions in the United Kingdom

**DOI:** 10.1111/nin.12512

**Published:** 2022-07-13

**Authors:** Iyore M. Ugiagbe, Liang Q. Liu, Marianne Markowski, Helen Allan

**Affiliations:** ^1^ Department of Adult Nursing, Child Nursing and Midwifery, Centre for Critical Research in Nursing and Midwifery Middlesex University London UK; ^2^ School of Health Sciences Faculty of Education, Health & Human Sciences, University of Greenwich London UK

**Keywords:** communication, discrimination, language competency, language test, overseas registered nurse, transition

## Abstract

Language tests for overseas registered nurses (ORN) working outside their home country are essential for patient safety, as communication competency needs to be established in any workforce. We argue that the current employment of existing language tests is structurally and institutionally racist and disadvantages ORNs from non‐European Union (EU) and non‐White countries seeking to work in the United Kingdom. Using Critical Race Theory (CRT), we argue that existing English language tests for ORNs seeking registration in the United Kingdom are discriminatory due to the UK's racist migration policies and a regulatory body for nursing and midwifery that fails to acknowledge and understand its own institutionally racist practices.

## 1 INTRODUCTION

Nurses and midwives are the largest professional group in the global healthcare workforce, and nursing shortages are a global concern (World Health Organisation, 2018). These shortages in global healthcare systems are predicted to continue (Scheffler & Arnold, 2019; Tuttas, 2015). Many western countries have recruited overseas registered nurses (ORNs) to work as registered nurses (Likupe, 2006) or as unregistered healthcare staff (Allan & Westwood, 2016a; Li et al., 2014; O'Brien, 2007) to address these shortages. Nearly 7% of the nursing workforce in the United States are ORNs, and in the United Kingdom, ORNs are 15.1% of the nursing workforce, either registered or working in an unregistered capacity in the healthcare sector (Organisation for Economic Co‐operation and Development [OECD], 2020).

We define ORNs as people trained to work as registered nurses in their home country who decide to work as nurses in a host country. Each host country has its approach to credentialing ORNs as fit to work as registered or licensed nurses based on a primary concern to maintain patients' safe care and working conditions for all staff. Any credentialing process is time‐consuming and expensive (Allan & Westwood, 2016b; O'Brien, 2007; Viken et al., 2018). Such processes may mean ORNs have to work as unregistered nurses in nursing assistant roles until they satisfy credentialing assessment. British National Health Service (NHS) employers actively recruit ORNs to work as Health Care Assistants (HCAs) (NHS Employers, 2021). This is an unofficial route to working in the United Kingdom but delays achieving language competency and registering with the British Nursing and Midwifery Council (NMC) (Allan & Westwood, 2016a). ORNs can spend many years working as HCAs seeking to register after failing repeated language tests (Allan & Westwood, 2016b).

ORNs must register with the NMC to work as registered nurses in the United Kingdom. The NMC requires ORNs to demonstrate that they can practice safely and effectively through a consideration of their qualification, training, and experience. To register, ORNs must show that they have the necessary knowledge of English to communicate effectively. These proofs can be evidenced in a number of ways which differ between non‐EU and EU‐trained ORNs. It costs £140.00 to register with the NMC to commence the initial registration process.

There are historical pushes and pulls to migration in the healthcare workforce globally (Kingma, 2007; Li et al., 2014; Viken et al., 2018). In the United Kingdom, overseas nurse recruitment was fundamental to establishing the NHS in 1948. The British Empire was the source for ORN recruitment in significantly large numbers in the early years of the NHS (McDowell, 2013; Snow & Jones, 2011). There were repeated waves of migration from the British Colonies and the extended Commonwealth (non‐European) countries to the United Kingdom, and nurses from colonial countries were not required to take a compulsory English language proficiency test.

Overseas recruitment of nurses to the United Kingdom was traditionally limited to former UK Colonies and Protectorates (Solano & Rafferty, 2007). However, in the 1970s, changes to the UK's global economic position led to increased calls for protectionism and reduced immigration amidst waves of antimigrant rhetoric and feeling (Snow & Jones, 2011; cited by Reynolds, 2019). Subsequent cycles of ORN migration have taken place in the context of racism toward migrants both in the NHS (Allan et al., 2004; Brathwaite, 2018; Smith et al., 2008) and in broader society (Reynolds, 2019; The Migration Observatory, 2020).

In 1971, following the UK's admission to the European Union (EU), the 1971 Immigration Act curtailed overseas nurse recruitment from the Commonwealth (Snow & Jones, 2011; cited by Reynolds, 2019); consequently, ORN recruitment from countries within the EU increased. Further changes in policy on overseas nurse registration and new conditions required for ORNs to gain admission to the NMC register (NMC, 2017a, 2017b) affected ORN recruitment from non‐EU and EU countries (Reynolds, 2019) as racist immigration policies continued (Gentleman, 2022). The number of ORNs registered in the United Kingdom doubled to 42,000 between 1999 and 2002. In 2001/2002, the NMC registered more ORNs than British‐educated nurses (Aiken et al., 2004). By 2015, EU‐trained ORNs comprised 4.5% of the NMC registered Nursing and Midwifery workforce compared with 8.2% from non‐EU countries (Maranzagov et al., 2016). The number of nurses from the EU registering with the NMC to work in the United Kingdom fell dramatically by 96% in 2017 after the Brexit referendum (Siddique, 2017). The recruitment of non‐EU ORNs continues to rise, with more nurses arriving in the United Kingdom than leaving to work overseas (Gillin & Smith, 2020).

Such immigration flows of nurses have always been contentious (Baxter, 1988; Brathwaite, 2018). Non‐White ORNs were viewed as outsiders and discriminated against by White ORNs (Allan et al., 2004; Baxter, 1988) in what is now recognised as institutional racism (Brathwaite, 2018). Recognition that the NMC was structurally and institutionally racist slowly emerged (Allan et al., 2004; Brathwaite, 2018; Smith et al., 2008; West et al., 2017). Despite these reports, criticism of racism within nursing has been muted (Hilario et al., 2017). A persistent colour‐blind approach, which is race and ethnicity neutral, has been practiced. This fails to acknowledge historical and contemporary racism in nursing (Allan et al., 2004; Brathwaite, 2018; Smith et al., 2008) and has implications for patient safety (Cunningham & Scarlato, 2018).

The paper discusses workplace challenges for ORNs, which arise from the current employment of English language testing in the recruitment of ORNs to work in the United Kingdom. Existing language requirements simply test competency in the English language but not the psychosocial skills required to integrate into the British workplace. We argue that the continued use of English language tests in the UK discriminates against ORNs from non‐EU, non‐White countries. They are evidence of structural and institutional racism against ORNs recruited to work in British health services. We argue that the tests are normalised in a professional discourse led by the NMC, which reproduces the historic and pervasive oppression of non‐White nurses in the NHS. These tests are used and misused to the detriment of the interests of non‐White ORNs seeking to register with the NMC to work in the United Kingdom.

### 1.1 Theoretical framing

Using Critical Race Theory (CRT), we suggest that English language tests for ORNs seeking registration in the United Kingdom are discriminatory and part of the UK's racist migration policies (Reynolds, 2019). The standardised tests for ORNs are not an adequate assessment of the professional language proficiency of applicants. These tests reveal processes of institutionalised racism, which operate in intricate and effective ways to prioritise Whiteness within nursing specifically and in health care more generally. For example, though an overall International English Language Test (IELTS) score of 7.0 is required for the ORN to commence the UK NMC registration process (NMC, 2017a), an IELTS score of 6.0 is required by fee‐paying international students to gain entry to a UK University such as Gloucester University (HCL Workforce Solutions, 2017). Additionally, an IELTS score of 6.5 guarantees admission to health‐related postgraduate courses in UK universities, while a score of 7.0 reserves admission to Warwick University, a Russell Group University, for MBA courses, yet, native English speakers have an IELTS average score of 6.9 (HCL Workforce Solutions, 2017), which is lower than the threshold score of 7.0 required for ORN to commence the UK NMC registration process.

CRT originated to examine, uncover and disrupt processes of racism in legal studies (Bell, 1995; Bryder, 1998). CRT is used increasingly in nursing to explore ideological, structural, and institutional racism (Ackerman‐Barger et al., 2019; Bennett et al., 2019; Brathwaite, 2018; Cunningham & Scarlato, 2018; Hilario et al., 2017). We understand structural racism to refer to the systematic exploitation of ethnic minority communities, which leads to material disadvantage; institutional racism relates to racism in institutional settings, political or social forces or influences, which reproduce racism through policies and practice, unconsciously or consciously. CRT illuminates colonial power and racism within the hierarchical and institutional structures of the NHS and nursing (Brathwaite, 2018). There are three separate strands to CRT:

(i) Racism is understood to be an everyday experience for people of colour, which is unacknowledged and challenging to address or eliminate. In not recognising or acknowledging racism, White people, as individuals and simultaneously as part of broader society, do not identify themselves as racist or part of a racist society (Clarke & Garner, 2010; DiAngelo, 2018).
(ii) CRT argues that White people's failure to see themselves as racist leads to a failure to see the interests that they accrue as Whites. These interests are economic, political, and psychological.
(iii) CRT views race as a social construct with no biological basis; individuals are defined in racial terms depending on those in power's economic and material interests (Diangelo, 2018).

In our choice of CRT, we go beyond a concern with recruitment ethics, which is a dominant discourse around ORN recruitment in both policy and nursing discourses. We explore the material disadvantages of language testing for ORNs who seek to work as registered nurses in the United Kingdom (Viken et al., 2018).

Our discussion focuses on language competency requirements, workplace challenges for ORNs, ORN recruitment practices, critiques of language tests, and the management of complaints against ORNs. The latter most frequently involve employers and colleagues complaining about language competency, including perceived difficulties in understanding ORNs' accents. We do not oppose language testing for ORNs whose nurse training was not in the English language to ensure communication competency. Our argument in this paper addresses a complex situation and encompasses several layers. First, we argue that the current use of these tests for NMC registration of ORNs trained in English is not fit for purpose. Second, we say that these standardised tests do not assess the professional language proficiency of applicants and are institutionally racist as they contain White cultural biases in their tasks and texts. Third, while employers have a stipulated responsibility to support ORNs as they transition into employment within the NHS, many ORNs struggle to meet the local, contextualised expectations of employers, coworkers, and patients/families once employed in the work setting. Fourth, those struggles may translate into complaints referral processes where ORNs frequently experience racist claims of communication failures. Fifth, some ORNs do not fully use their educational and professional expertise; they are often paid at a much lower rate than the positions they initially apply for or are qualified for and are likely to remain in these positions if they are not supported in passing the language requirements and NMC registration (Allan & Westwood, 2016a; Smith et al., 2008).

### 1.2 Language tests

Compulsory language tests were first introduced in America in the 1970s (Mueller, 2016), followed by Australia in 2000 (Wickett & McCutcheon, 2002) and the United Kingdom in 2005 (NMC, 2017a). ORNs from outside the European Economic Area (EEA) must take either the IELTS or the Occupational English Test (OET). In 2007, the IELTS minimum score level was increased from 6.5 (competent) to 7.0 (Good) across the four test modules of reading, writing, speaking, and listening in the United Kingdom. Raising the score level from 6.5 to 7 resulted in many ORNs not achieving the required test result despite working competently as HCAs in the United Kingdom (NMC, 2017c, 2017d). In 2016, the NMC extended the IELTS requirement to nurses within the EU/EEA (HCL Workforce Solutions, 2017; NMC, 2017b; Royal College of Nursing, 2017). In November 2019, the NMC lowered the written assessment part of IELTS to 6.5 to allow more ORNs to pass but maintained level 7 for speaking, listening, and reading (Gilroy, 2019).

Before 2016, the NMC accepted only IELTS. Since 2017, the NMC has accepted three language competency types as evidence, evaluated case‐by‐case. The first evidence is for ORNs and midwives to demonstrate that they have attained level 7 in IELTS or level B in the OET. The second form of evidence is a completed preregistration nursing or midwifery programme taught and examined in English. The third form of proof is when the international nurse or midwife can demonstrate that they have practiced for one  year in a country where the English language is the first and native language (NMC, 2017c, 2017d). ORNs would only be required to prove language skills again if the particular ORN was referred to the NMC in a fitness to practice (FfP) case because the employer or patients had raised concerns about their ability to communicate in English.

In 2016, only nurses from the EEA could provide suitable evidence of their ability to communicate in English or pass the IELTS at level 7.0 (Royal College of Nursing, 2017). There was no reason for excluding non‐EEA countries where primary, secondary and pre‐registration nursing education is delivered in English (NMC, 2017e). We argue that compiling a list of countries considered to have English as a first and native language but excluding African countries such as Nigeria, Ghana, Kenya, Zimbabwe, and South Africa, where primary, secondary, and nursing education is in the English language, is evidence of institutional racism.

The British Council, International Development Program Education, and Cambridge English Language Assessment developed IELTS for people who intend to study or work where English is the primary language. The test fee varies depending on the individual testing centre and ranges between £150 and £200 (Takeielts.britishcouncil.org, 2018). IELTS do not assess communication in nursing practice or social‐pragmatic competence (Sedgwick & Garner, 2017). The IELTS academic assessment for nurses and midwives was criticised for focusing on intellectual and academic abilities (Lynch, 2016). In 2017 the NMC undertook a consultation that recommended widening language tests to recognise the social context of language (NMC, 2017e). The social context of language includes ORNs' ability to respond effectively in clinical situations and their familiarity with cultural knowledge and local colloquialisms (Smith et al., 2008) to ensure safety in clinical workplaces for patients and staff.

The OET was developed in the 1980s by the Australian National Office for Overseas Skills Recognition (NOOSR). It has been used for immigrant healthcare workers for over 30 years. The OET differs from the IELTS by offering communication scenarios candidates are likely to meet in healthcare. It assesses the language competency of ORNs in situ rather than simply academically as the IELTS does. The OET still falls short in authentic representations of real‐world interactions because it does not give contextual information to candidates or test takers (Woodward‐Kron & Elder, 2016). The cost for OET (£340) is higher than the price of IELTS (£140). During an NMC consultation on the English language (NMC, 2017c), the higher cost of the OET was seen as a potential barrier for employers and ORNs, especially for ORNs from low‐income countries (Allan & Westwood, 2016a).

#### 1.2.1 Are language tests reliable and valid?

The language test organisations have checked both OET's and IELTS' internal reliability and validity using Cronbach's *α*, which measures internal consistency. A Cronbach's *α* coefficient of 0.90 or above is generally considered excellent (Gliem & Gliem, 2003). In 2017, the IELTS listening sections had an overall Cronbach's *α* of 0.91, the general training reading was 0.92, and the academic reading was 0.90 (IELTS, 2018). In 2019, the reliability of each subtest for OET was reported as Cronbach's *α* of 0.79 in the listening subtest, reading subtest was 0.80, writing subtest was 0.79, and speaking subtest was 0.77. OET seems to have lower internal reliability than IELTS, though some statisticians have raised concerns that a very high value implies redundancy in test items (Tavakol & Dennick, 2011).

Concerns around IELTS' and OET's generalisability about representativeness and relevance of the construct and meaningfulness of interpretations are evidenced by previous research (Hamp‐Lyons, 1990; Uysal, 2010; Woodward‐Kron & Elder, 2016). These include lack of representation of real‐world interactions, the bias of single marking of papers, readability of prompts, and comparability of writing topics. For instance, Hamp‐Lyons (1990) argues that elements of the writing assessment in IELTS (the writer, task, and raters) and the scoring procedure are sources of error that reduce the reliability of this aspect of the test. IETLS assess writing skills on topics or contexts of language use developed from British and Australian cultural contexts, which introduces a bias against candidates of other backgrounds. It is acknowledged that controlling the topic variable is not an easy task. It is challenging to determine a joint knowledge base that all students can access from culturally diverse backgrounds (Kroll & Reid, 1994). Writing is single marked locally, and rater reliability is estimated by subjecting a selected sample of returned scripts for second marking by a team of senior examiners (Shaw, 2007). In an international test, a single marking is inadequate. In writing assessment, it is widely accepted that multiple judgements lead to a final score closer to a correct score than any judgement (Hamp‐Lyons, 1990; Uysal, 2010). In addition, the OET speaking subtest roleplay performances of 12 doctors who were successful OET candidates were compared with the practice Objective Structured Clinical Examination (OSCE) roleplay performances of 12 international medical graduates by Woodward‐Kron and Elder (2016). For various reasons, they found that both tests fall short in the authentic representation of real‐world interactions, mainly the OET task. These include time allowances, training of test interlocutors, and the limits of contextual information provided to candidates, which constrains candidate topic exploration and treatment negotiation. Given the importance of ‘real world’ communication and the topic variable on writing performance and the difficulty of controlling it in an international context, the comparability and appropriateness of both IELTS and OET are questionable.

Regarding language test validity, although IELTS claims to have adopted ‘international English’, the IELTS test questions were developed by writers in Australia, Canada, New Zealand, the United Kingdom, and the United States and refer to ‘native’ varieties of English (Hamid et al., 2019).

Concerns about the validity and reliability of international language tests are reported in the literature (Freimuth et al., 2016; Gagen, 2019; Taylor, 2002). These relate to cultural biases and tests being constructed from a White standpoint. In a study conducted in Bangladesh on the cultural bias of the speaking examination, it was found that minimising test bias became the examiner's responsibility, not the examination itself (Khan, 2006). Examiners had to avoid the topic of discussion that was unfamiliar to test‐takers. Another study found that in 27% of the 572 countries surveyed, IELTS test‐takers perceived ‘unfairness’, with almost a third of the candidates interviewed posttest indicating a concern with the cultural bias of topics and materials on the IELTS (Hawkey, 2005). In a content analysis study of IELTS, it was found that cultural bias was embedded in IELTS. This bias was perceived by United Arabic Emirate students and attributed to sociocultural and educational background differences from western countries. Items of ‘cultural concern’ for Emirate students included essay topics on the right to freedom of speech for artists and volunteer work, which are not as widely known in United Arabic Emirate society as in Western societies (Freimuth, 2016). In terms of predictability, Gagen performed a meta‐analysis of 18 studies examining how IELTS scores used for admission relate to actual student scores once in postsecondary education. They conclude that the IELTS test has a small predictive effect on performance in postsecondary programmes (Gagen, 2019).

The validity of test results is challenged by the lack of inclusion of social and regional language variations in test input in terms of content and linguistic features without considering variations among rhetorical conventions and genres worldwide, such as including various cultures. And accents are not being considered in either IELTS or OET (Abidin & Jamil, 2015; Freimuth et al., 2016; Taylor, 2002).

## 2 WORKPLACE CHALLENGES FOR ORN

Another substantial criticism of both IELTS and OET is that, despite ORNs passing the language test, communication barriers and cultural differences remain common challenges for them when working after registration with the NMC in the United Kingdom (Mueller, 2016; NMC, 2017e; Stubbs, 2017; Woodward‐Kron & Elder, 2016). Some ORNs experience difficulties with the language in clinical workplaces despite passing the language tests (O'Neill, 2011). This suggests that several social and workplace factors need to be in place before an ORN is proficient, even after language testing.

In subsequent studies (Allan et al., 2004; Allan, 2007; Allan & Westwood, 2016a; Bachman, 1990; O'Neill, 2011; Xiao et al., 2014), interviews with ORNs show that they struggled with communication and conversation with patients and colleagues as their language competency test had not assessed their English for the clinical setting. To address deficiencies in workplace language competency, ORNs develop practical skills, including a masterful grasp of English rules to acquire language competency (O'Neill, 2011). Variations between native and host countries in nursing practice, besides communication difficulties with patients, present a further challenge for ORNs' integration into the workforce (Likupe, 2006; Lum et al., 2015; Smith et al., 2008). Pung and Goh (2017) show that ORNs face challenges at every integration stage, from registration to settling into local communities and developing a career. The same authors argue that language obstacles result in ORNs working less efficiently, which increases workplace stress for ORNs as they attempt to integrate into their host countries. Pung and Goh conclude that ORNs feel scapegoated by their mentors when they make mistakes and that blame for these mistakes is attributed to their 'poor' language skills.

Smith et al. (2008) found that ORNs were deskilled and failed to progress in their careers due to deep‐rooted direct and indirect discriminatory practices involving racist beliefs about ORNs' competencies in English. Language incompetence was often ascribed to ORNs' accents, and British workers failed to accept different accents in ORNs. ORNs described struggling to master local dialects and colloquialisms. During the initial transition to the British workplace, mentors were significant figures in maintaining racist discourses about ORNs' perceived lack of competency in English. In response to these discriminatory practices, ORNs became unwilling to publicly expose themselves to ridicule for team meetings or telephone conversations with relatives (Allan et al., 2009). Their unwillingness to answer the ward telephone particularly became further evidence for the British coworkers of their language incompetence (Smith et al., 2008).

Such findings align with an integrative literature review of ORNs' experiences and socioprofessional integration cross‐culturally (Primeau et al., 2014). The authors identified obstacles to ORNs' professional integration and critical strategies for their successful integration into professional practice in the host country. Six major blocks for ORN professional integration included: the recognition of skills and experience, differences in nursing practice, differences in technologies, communication barriers, cultural differences and discrimination by team members, managers and patients.

Kawi and Xu (2009) also showed that differences in pronunciation, accent and terminologies led to ORNs being perceived as having inadequate language skills. ORNs faced challenges in understanding the sociocultural aspects of communication as in‐jokes, sarcasm, euphemisms and nonverbal behaviours; failing to understand the nuances of culturally appropriate language and a lack of support and inadequate orientation to the culturally appropriate language. Kawi and Xu (2009) argue that a positive work ethic, persistence, psychosocial and logistical support, learning to be assertive and continuous learning facilitated the adjustment of ORNs to their new workplace environments. These strategies all helped language acquisition and were not ‘tested’ in language tests.

To understand the intercultural communication experiences and associated communication training needs of ORNs in the Australian healthcare system, Philip et al. (2015) conducted an exploratory qualitative study. Interviewing nurse educators, three key communication challenges were thought to face ORNs on arrival in Australia: barriers to intercultural communication, for example, nurses' reluctance to engage in communicative strategies that build rapport with patients; transitional behaviours and their impact on communication, including maintenance of perceived cultural hierarchies between health professionals; development of communicative competence. They concluded that communication is not merely a skill taught in a traditional 'chalk and talk' teaching programme. A comprehensive understanding is needed of the sociocultural dimensions of ORNs' orientation as they transition into work in the host country. Poor transition experiences grounded in poor communication can affect how ORNs learn to communicate in new healthcare settings and how their co‐workers accept them.

Lum et al. (2015) argue that achieving adequate communication proficiency is a long‐term, multistage process. This process must also incorporate understanding and acquisition of the local host society's social and cultural aspects. Communication skills cannot be taught or assessed by didactic teaching programmes or single‐point language tests.

## 3 RECRUITMENT AND TRANSITION TO PRACTICE IN THE UNITED KINGDOM

The responsibility for the successful transition of ORN from donor to host country lies with the employer (NHS Employers, 2017). If it becomes apparent once appointed that the nurse's communication skills are not at the required level, it is the employer's responsibility to support the individual in gaining the appropriate language and communication skills (NHS Employers, 2017). Some NHS trusts have successfully helped ORNs with language teaching support (NHS Employers, 2016). However, this support is generally uneven. In privately run care homes, the employer's support for acquiring the necessary English level may be inadequate or nonexistent (Allan & Westwood, 2016b). NHS trusts outsource their recruitment (Gillin & Smith, 2020) to agencies which do not apply accepted ethical standards and, thus, reproduce discriminatory practices (Martin, 2017).

This paper shows that language tests do not assure language readiness to communicate with colleagues within the multidisciplinary teams and patients in the clinical workplace (Lum et al., 2015). Mandatory language tests with strict score requirements at the advanced academic level may not be appropriate for ORNs as they transition to a foreign workplace and learn to communicate effectively for patient safety in the host country's healthcare system. Preparation for the existing tests seems not to focus on skills other than those needed to pass the test. Despite ORNs passing the language test, communication barriers and cultural differences remain common challenges when working after registration with the NMC in the United Kingdom and Australia (O'Neill, 2005).

ORNs often express surprise at the magnitude of adjustment required to adapt to working in a new foreign environment after showing language competency at testing (O'Neill et al., 2005). The language journey from the classroom to the clinical setting is a process that goes beyond the notions of language proficiency. Rumsey et al. (2016) found that all participants in the study indicated concerns with the suitability of the IELTS as a test system. The test was not relevant to their work, requiring several test sittings. The tests themselves are not fit for purpose, but their proof of language competency is also flawed (Mueller, 2016); the seemingly arbitrary threshold changes cause confusion and anxiety for ORNs (Allan & Westwood, 2016a, 2016b).

## 4 THE MANAGEMENT OF COMPLAINTS AGAINST ORNS

These critiques of language testing need to be seen in the context of reported structural and institutional racism in British health services (Adhikari, 2020; Alexis & Shelling, 2015; West et al., 2017). Smith et al. (2008) concluded that complaints of competency are not objectively processes. They are open to interpretation and framed by institutional racism. Complaints about ORNs are a complex social process that frequently emerges from the failure to integrate ORN into NHS teams and support them with social language skills acquisition (Allan & Larsen, 2003; Smith et al., 2008). While a large number of non‐White ORNs are referred to the NMC for an FfP review, Smith et al. (2008) concluded after interviews with NMC staff that the NMC had little understanding of racism and perceived their FfP procedures to be colour‐blind. Complaints of ORNs' practices are compounded by employers' and the NMC's colour‐blind approach, their belief that the ethnicity of the ORN or Black member of staff is unrelated to the frequency of referral to FfP. Analysis of NMC FfP transcripts by West et al. (2017) shows that such complaints result from poor teamwork and poor integration of nurses from Black, Asian and Minority Ethnic (BAME) and ORN/BAME backgrounds. These practices around complaints are further evidence of institutional racism in British health services and nursing. Institutional racism is a daily part of ORNs' working lives in the NHS (Allan & Larsen, 2003; Larsen et al., 2005; Smith et al., 2008). ORNs experience direct or indirect discrimination when employed, being refused employment as a registered nurse or being offered work as a lower‐paid healthcare worker (Smith et al., 2008). Allan and Westwood (2016b) showed that ORNs in London remained in lower‐skilled and lower‐paid work for many years trying to gain their IELTS 7 score across all test domains yet repeatedly failing through lack of support. Repeated attempts come at a financial cost. Once recruited, one should note that the employers' responsibility is to ensure that the ORN in question is given the appropriate support to improve their language skills by transitioning into working in the United Kingdom (NHS Employers, 2017). However, ORNs complain that employers frequently fail to honour this commitment.

West et al. (2017), in their analysis of referrals to and progress through NMC FtP review data, found a nurse's ethnicity determined referral, source of referral and outcome in unexpected ways. African heritage nurses and midwives and those of unknown ethnicity are disproportionately represented in referrals to the NMC; African heritage male nurses are more likely to be referred to the NMC than White male nurses. Non‐White nurses and midwives are disproportionately represented in referrals by employers, whereas White nurses and midwives are disproportionately represented in referrals by members of the public. The likelihood of referral is consequential in terms of progress and outcomes of the FtP process. While BAME staff are more likely to be referred, they are also more likely to have no case to answer. White nurses are less likely to be referred but are more likely to have more severe charges to respond to and receive a severe sanction (West et al., 2017).

## 5 DISCUSSION

We have argued that the language tests used to recruit non‐EU ORNs to work in the UK instantiate racist ideologies. We have shown that the language tests themselves have biases, poor reliability and validity, and do not support the assessment or development of sociocultural competence of ORNs, which are needed for safe practice as ORNs settle into the United Kingdom. These biased tests are then used to register ORNs with the NMC, which fails to recognise its part in a racist recruitment system. Ultimately, existing language tests position BAME ORNs from the Commonwealth at a disadvantage in terms of employment and workplace, where ORNs face racism and are unsupported by employers. The system of language testing, the tests themselves, and the support subsequently offered to ORNs to settle in the United Kingdom as invited workers are evidence of racism at the heart of both the NMC and the home office, which fail to acknowledge the limitations of the tests and the context in which they are used (Gentleman, 2022).

We have shown how current language tests disadvantage ORNs from non‐EU, non‐White countries in ways that prioritise White EU ORNs. Language testing controls and selects who can enter and practice as ORNs in the United Kingdom in ways that reproduce historical racist ideologies in British employment and migrant policies (Reynolds, 2019). Language tests are exclusionary strategies that produce exclusionary and racist immigration policies (Reynolds, 2019). This racist ideology has become more influential in immigration policy since the Brexit vote in 2016 (Allan, 2016) and particularly so since the 2019 UK general election (Reynolds, 2019). These tests control who enters the United Kingdom to work as an ORN in complex ways, including payment for the testing, an individual burden that privileges ORNs who have social support to pay for the initial and then repeated tests. The possibility and encouragement of the unregistered route to work in the United Kingdom mean that ORNs find poorly paid work in the United Kingdom in NHS trusts which exploit their labour while pushing the financial burden of repeated tests onto the ORN. Furthermore, when in 2016, nurses from EEA had the alternative to provide suitable evidence of ability to communicate in English or pass the IELTS at level 7.0 (Royal College of Nursing, 2017); there was no justifiable rationale for the exclusion of African countries (Nigeria, Ghana, Kenya, Zimbabwe and South Africa) where primary, secondary and preregistration nursing education is in the English language. Excluding these countries is institutionally racist and privileges Whiteness and White supremacy.

As well as acting as barriers to qualified ORNs who seek to register with the NMC in the United Kingdom, language tests are insufficient proof of language competency in the workplace. Passing the test does not prepare ORNs for integration into workplaces; rather, it exposes them to racism, discrimination, exclusion and exploitation. Bias arises from racist beliefs about ORNs' competencies in English and a failure among British workers to accept difference and diversity in colleagues. This further institutionalises White privilege and White supremacy both within nursing and healthcare generally (Blanchett Garneau et al., 2018) and is seen by Reynolds (2019) as protective of what has historically and ideologically been seen as a ‘besieged Island nation’.

We conclude that IELTSs and OETs for ORNs seeking registration in the United Kingdom are discriminatory due to British racist migration policies, institutional racism in the NHS, and a regulatory body that fails to understand the impact of racism on its practices. We believe that such racism, based on a White supremacist perspective of the world and how it works, arises from an uncritical understanding of how ethnicity and racism shape and constrain ORNs' lives (Lammy, 2020). A glaring example is the exploitation of ORNs who face expensive, repeated language testing while working in assistant nursing roles. An effect of this repeated testing is the demoralising effect failure has on these ORNs (Allan & Westwood, 2016b). Another example is the exposure to the racism that ORNs face in the workplace after passing the language tests because preparation for these tests does not prepare them for clinical workplaces (O'Neill, 2011). Current language preparation guidance and testing are colour‐blind and ignore that the language journey from the classroom to the clinical setting is a process that goes beyond the notions of language proficiency and starts with an ORN's ethnicity and access to material resources (Lum et al., 2015) which discriminates against BAME ORNs. Mandatory language tests with strict score requirements at the advanced academic level are inappropriate because they divorce language acquisition from its social context and assume a language test implies an ability to communicate in the workplace.

Consequently, even where trusts support ORNs to prepare for the tests, support is not ongoing. The high number of complaints to the NMC FfP review is unsurprising given the difficulty of acquiring language competency and fluency and the lack of willingness among host employees and managers to tolerate differences in language competency. The NMC's failure to acknowledge its institutional racism is a sad indictment of British nursing's deeply entrenched institutional racism (Brathwaite, 2018).

Setting language competency tests/requirements that are colour‐blind and based on a narrow definition of language competency suggests evidence of a colour‐blind and culture‐blind approach to tests. It appears to us that the dominant group's White supremacist belief that language is purely an intellectual achievement rather than constructed by social habitus and access to resources is fundamentally racist. An unwillingness to acknowledge unconscious bias in nursing and the daily experiences of ORNs as they face racism because of language differences and lack of fluency arises from nursing's historical racism and unwillingness to examine the fallacy of the White liberal viewpoint (Allan, 2021).

## 6 CONCLUSIONS

Health systems can only function with sufficient health workers who work collaboratively. National and community‐based expectations for countries to put recruitment and employment procedures to ensure safe, competent, and ethical care are stated in the WHO's Global Code of Practice on the International Recruitment of Health Personnel (World Health Organisation, 2010). If the recruitment is well‐managed, the international migration of nurses can contribute to developing and strengthening healthcare systems.

With the UK's exit from the EU, it is timely and appropriate for the NMC to consider introducing localised workplace and appropriate language assessments as part of the employing organisation's recruitment process fairness and equity. This would help reduce the recruitment challenges that have faced the NHS employers for several years. This measure will encourage flexibility and commitment to improving the overseas nurse's communication skills in clinical practice. Healthcare employers need to ensure that ORN recruitment is inclusive, antiracist and ethical even when outsourced. After recruiting ORNs, the employer must provide a suitable system to help them settle into their host country. This system needs to address cultural and corporate practices and give specific language support even when language tests are passed. We argue that while the inherent purpose of language tests is to provide a test for competency for clinical practice, existing language tests are not always the most effective tool to assess competency or predict integration at work in countries which recruit ORNs and other overseas‐trained professionals. In most Western countries, nursing regulatory bodies are responsible for evaluating, training and updating ORNs' nursing competencies to ensure they are ready to be integrated into the healthcare system. We argue that further work needs to be undertaken by the NMC to ensure language tests are fit for purpose.

However, these actions are not enough to tackle racism in nursing. Nurses must adopt critical approaches (including CRT and postcolonial feminism) to explore racism in nursing.

## CONFLICT OF INTEREST

The authors declare no conflict of interest.

## Data Availability

Data sharing does not apply to this article as no datasets were generated or analysed during the current study.
